# Samaritan Free Hospital for Women and Children

**Published:** 1895-04-13

**Authors:** 


### THE INSTITUTIONAL WORKSHOP.

### WITHIN THE HOSPITALS.

### SAMARITAN FREE HOSPITAL FOR WOMEN AND CHILDREN.

THE BUILDING.

Passers along that dullest of thoroughfares, the Marylebone Road, must know well the cheerful red-brick building which bears across its front the legend, "Samaritan Free Hospital for Women and Children." Situated on the south side of the road near the Paddington end, it is within convenient reach of Edgware Road and Baker Street Stations, and standing well back from the road and somewhat above its level, is as much protected from noise as a building in a London street may be. The brightness of its many-windowed front is not belied by the interior. Passing up the high flight of steps to the entrance, the visitor finds himself in a spacious hall, open to the roof, well ventilated and lighted. In the basement are the kitchen offices, which are ample and convenient, linen-room, &c.; on the ground floor are the board-room, secretary's office, matron's sitting-room (a cheerful little apartment), nurses' dining-room, dispensary, and three wards; on the first floor, several wards, matron's bed-room, operating theatre, &c.; the second floor contains seven wards and a room for two nurses; and the third several single-bed wards, medical and surgical wards, and nurses' bed-rooms. Lavatories and bath-rooms are in two projecting wings at the back of the building, one wing being for the medical and the other for the surgical side, the two being separated from the main building by cross-ventilated lobbies. The two divisions of the hospital are kept entirely distinct. The surgical side is naturally specially arranged for the cases of serious operation for which it is intended. The single bedded rooms kept for such cases are particularly cosy-looking, and those on the south side on bright days form veritable sun-baths. It is unfortunate that the position of the building is such as to face north and south, so that those wards on the north or front side practically get no sunshine, but barring this defect nothing can exceed the pleasant and home-like aspect of all the wards in the building. They are heated by open fires. On the medical side there is a large, cheerful "convalescent ward," with prettily decorated walls, and well supplied, as, indeed, are all the wards throughout, with pictures and plants.

HISTORY OF THE HOSPITAL.

The history of this hospital is not without a special interest of its own. As intimately connected with the splendid work of Sir Spencer Wells, it has attained an almost unique reputation in its particular field of surgical practice, and has led the van in the successful treatment of cases where operation not so many years ago was denounced as "akin to murder." The Samaritan Hospital originated some fifty or so years since, when Dr. William Jones "hired a small room at a few shillings a week, which he destined should be the forerunner to a special hospital for women. He not long afterwards transferred himself and patients to a general dispensary. Believing still that a hospital of a more decided character was called for and would be supported, Dr. Henry Savage rented a house in Orchard Street. Dr. Jones came over to it. A few beds were eventually provided, and thus commenced hospital work at all warranting the title; the one adopted, after some hesitation over the word Samaritan, being—The Samaritan Free Hospital for Diseases Peculiar to Women and Children. Admission free. No letter of recommendation required. Poverty and sickness the only passport." (The quotation is from a small publication containing "Some Leading Incidents in the History of the Hospital.") After a time, and owing to differences of opinion, Dr. Jones retired, and later Dr. Savage was joined by Dr. Routh and Mr. Wells. At this time the operation of ovariotomy was almost generally attended by fatal results. The then consulting physician was averse to the admission of these cases, though it was from the first warmly urged by Mr. Wells, and the coroner declared he would hold an inquest over every fatal case. "In 1858, however, Mr. Wells was permitted to operate on three ovarian cases," they were all successful, and from this time forward the record, not only of these cases, but of other serious abdominal operations, has been yearly increasingly triumphant. The proportionate mortality beginning at one in three has steadily decreased till at the present time it is little over 5 per cent. After these early days in Orchard Street the hospital was moved to Seymour Street, and here four new wards were built on the top of the house, and an efficient out-patient department provided. In 1889 the stone of the present building was laid by H.R.H. the Prince of Wales, and it was opened for patients in 1890.

ADMINISTRATION.

The economy of its general management was singled out for especial commendation last year in "The Hospital Annual," in noting a "further reduction at the Samaritan Free Hospital, where the percentage of cost of administration to maintenance has fallen from 14·723 in 1891, a comparatively low percentage, to 14·576 in 1892." Continuing, the "Annual" says, "The percentage of cost of administration to total ordinary expenditure at the Samaritan Free Hospital is only 12¾ per cent., a fact which proves Mr. George Scudamore to be one of the ablest of hospital economists. . . . The institution has had many and great difficulties to contend with; and, as we remarked on a former occasion, it is not too much to say that the results shown by the figures . . . . bear eloquent testimony, not only to the efficiency of Mr. Scudamore, but to the care which he exercises in the administration of one of the best managed of special hospitals." That this desirable result is not arrived at by a too rigorous cutting down of expenditure is abundantly testified by the ample provision for the comfort of patients, the dainty appurtenances of the wards, and the excellence of the dietary.

A SUGGESTION.

It may readily be imagined that for many of the patients admitted to such a hospital as this, possibly to undergo some terrible operation, an after change, before a return to the home, is all but a necessity; yet beds at convalescent homes are by no means always obtainable. There is a great field for useful help from the rich in this especial particular, and the hint thrown out in a late report that "It is often possible for a lady in some quiet rural spot to hire a room in a cottage or farm, where, for a very few shillings a week, a poor woman could be housed, and thus have rest, fresh air, and simple nourishing diet, which would firmly establish the good done at the hospital," might well be acted upon more frequently by those interested in the Samaritan Free Hospital and its work. For a comparatively small expenditure many a poor sufferer might thus be helped to health and strength before starting afresh upon the battle of life. Some assistance in this way is already given by a few kind friends, but it is needless to say more would be thankfully accepted.

The Samaritan Free Hospital fulfils a useful mission, and those who have any doubt on the subject should go and see Miss Butler, the matron, who would, we feel sure, gladly extend to them the courtesy shown to the writer in taking them over the building and explaining its work and its needs. Money is now wanted for the provision of a better out-patient department, the present premises being very inadequate, and it is much to be hoped that this very necessary improvement may soon be effected. This hospital has a special claim upon country subscribers, for the number of patients from outside London treated yearly within its walls is very considerable. As its name implies, admittance is entirely free.

